# A Text-Book of Physiology, Normal and Pathological

**Published:** 1906-01

**Authors:** 


A TEXT-BOOK OF PHYSIOLOGY, NORMAL AND PATHOLOGICAL For Students and Practitioners of Medicine. By Winfield S. Hall, Ph.D. (Leipzig), M.D. (Leipzig), Professor of Physiology, Northwestern University Med. School, Chicago; Member of the Physiological Society; Chairman of the Section of Pathology and Physiology, American Med. Association, 1904–5; Fellow American Academy of Medicine, etc. Second edition, revised and enlarged, 340 engravings and 3 colored plates. Lea Bros. & Co., Philadelphia and New York.

Notable additions to the second edition of this work are the subchapters on Pathologic Physiology. This enables the physician to better understand the diseased conditions and appreciate the variations from normal. All the important laboratory experiments are clearly described. The engravings are excellent and bring out lucidly the points they were designed to portray.

